# Supplementary material for: Modeling glioblastoma heterogeneity as a dynamic network of cell states
Source: Mol Syst Biol. 2021 Sep 16;17(9):e10105. doi: 10.15252/msb.202010105 (PMC8444284; doi:10.15252/msb.202010105)
Supplement: Supplementary file 6 — Source Data for Figure 5 [file MSB-17-e10105-s004.zip › Figure5A_sourcedata/GSEA_3071/nosigdb_stateV.GseaPreranked.1622795159954/VANGRONINGEN_ADRN.html]

Details for gene set VANGRONINGEN\_ADRN[GSEA]

|  || Dataset | state43071 |
| Phenotype | NoPhenotypeAvailable |
| Upregulated in class | na\_pos |
| GeneSet | VANGRONINGEN\_ADRN |
| Enrichment Score (ES) | 0.5413653 |
| Normalized Enrichment Score (NES) | 3.2593203 |
| Nominal p-value | 0.0 |
| FDR q-value | 0.0 |
| FWER p-Value | 0.0 |
Table: GSEA Results Summary

  

Fig 1: Enrichment plot: VANGRONINGEN\_ADRN      
 Profile of the Running ES Score & Positions of GeneSet Members on the Rank Ordered List

  

| PROBE | GENE SYMBOL | GENE\_TITLE | RANK IN GENE LIST | RANK METRIC SCORE | RUNNING ES | CORE ENRICHMENT || 1 | NUSAP1 |  |  | 6 | 2.001 | 0.0119 | Yes |
| 2 | CCP110 |  |  | 9 | 1.920 | 0.0241 | Yes |
| 3 | ASRGL1 |  |  | 16 | 1.819 | 0.0349 | Yes |
| 4 | CKB |  |  | 23 | 1.748 | 0.0452 | Yes |
| 5 | RRM2 |  |  | 34 | 1.660 | 0.0541 | Yes |
| 6 | MYBL2 |  |  | 35 | 1.651 | 0.0649 | Yes |
| 7 | CENPU |  |  | 36 | 1.645 | 0.0757 | Yes |
| 8 | ASCL1 |  |  | 43 | 1.604 | 0.0850 | Yes |
| 9 | GNG4 |  |  | 47 | 1.590 | 0.0949 | Yes |
| 10 | KIF15 |  |  | 52 | 1.568 | 0.1044 | Yes |
| 11 | FOXM1 |  |  | 53 | 1.550 | 0.1145 | Yes |
| 12 | PBK |  |  | 54 | 1.536 | 0.1246 | Yes |
| 13 | GAP43 |  |  | 61 | 1.501 | 0.1333 | Yes |
| 14 | INSM1 |  |  | 72 | 1.467 | 0.1409 | Yes |
| 15 | NRCAM |  |  | 75 | 1.449 | 0.1500 | Yes |
| 16 | UBE2T |  |  | 79 | 1.432 | 0.1588 | Yes |
| 17 | KLHL23 |  |  | 83 | 1.421 | 0.1675 | Yes |
| 18 | MCM7 |  |  | 88 | 1.400 | 0.1759 | Yes |
| 19 | HES6 |  |  | 91 | 1.391 | 0.1847 | Yes |
| 20 | OLFM1 |  |  | 102 | 1.361 | 0.1916 | Yes |
| 21 | CXADR |  |  | 103 | 1.360 | 0.2005 | Yes |
| 22 | NCAM1 |  |  | 117 | 1.334 | 0.2067 | Yes |
| 23 | ZNF704 |  |  | 120 | 1.329 | 0.2151 | Yes |
| 24 | CDCA5 |  |  | 123 | 1.326 | 0.2233 | Yes |
| 25 | CELF2 |  |  | 137 | 1.300 | 0.2293 | Yes |
| 26 | PKIA |  |  | 138 | 1.297 | 0.2378 | Yes |
| 27 | GMNN |  |  | 146 | 1.280 | 0.2448 | Yes |
| 28 | TUBB2B |  |  | 148 | 1.278 | 0.2530 | Yes |
| 29 | CADM1 |  |  | 152 | 1.264 | 0.2607 | Yes |
| 30 | CDKN3 |  |  | 163 | 1.239 | 0.2669 | Yes |
| 31 | RFC4 |  |  | 171 | 1.227 | 0.2736 | Yes |
| 32 | DPYSL5 |  |  | 178 | 1.218 | 0.2804 | Yes |
| 33 | ATP6V0E2 |  |  | 181 | 1.209 | 0.2879 | Yes |
| 34 | GGH |  |  | 182 | 1.207 | 0.2958 | Yes |
| 35 | NUF2 |  |  | 192 | 1.189 | 0.3018 | Yes |
| 36 | TCEAL7 |  |  | 200 | 1.171 | 0.3082 | Yes |
| 37 | IGSF3 |  |  | 206 | 1.165 | 0.3148 | Yes |
| 38 | RBBP8 |  |  | 210 | 1.162 | 0.3218 | Yes |
| 39 | PRC1 |  |  | 211 | 1.161 | 0.3294 | Yes |
| 40 | ARL6IP1 |  |  | 212 | 1.158 | 0.3370 | Yes |
| 41 | UBE2C |  |  | 214 | 1.154 | 0.3444 | Yes |
| 42 | KIF21A |  |  | 217 | 1.151 | 0.3516 | Yes |
| 43 | CDKN2C |  |  | 223 | 1.143 | 0.3581 | Yes |
| 44 | MCM2 |  |  | 238 | 1.130 | 0.3627 | Yes |
| 45 | DCX |  |  | 258 | 1.107 | 0.3663 | Yes |
| 46 | KIF5C |  |  | 261 | 1.102 | 0.3731 | Yes |
| 47 | MAP2 |  |  | 262 | 1.100 | 0.3803 | Yes |
| 48 | MYEF2 |  |  | 271 | 1.090 | 0.3859 | Yes |
| 49 | MCM6 |  |  | 282 | 1.080 | 0.3910 | Yes |
| 50 | BEX1 |  |  | 287 | 1.077 | 0.3973 | Yes |
| 51 | PRIM1 |  |  | 293 | 1.074 | 0.4033 | Yes |
| 52 | H1FX |  |  | 310 | 1.055 | 0.4071 | Yes |
| 53 | PHYHIPL |  |  | 367 | 0.987 | 0.4026 | Yes |
| 54 | DNAJC9 |  |  | 373 | 0.979 | 0.4081 | Yes |
| 55 | ALK |  |  | 399 | 0.951 | 0.4094 | Yes |
| 56 | DPYSL2 |  |  | 419 | 0.935 | 0.4118 | Yes |
| 57 | VRK1 |  |  | 445 | 0.911 | 0.4129 | Yes |
| 58 | EML6 |  |  | 446 | 0.910 | 0.4188 | Yes |
| 59 | KNSTRN |  |  | 457 | 0.902 | 0.4228 | Yes |
| 60 | ZNF738 |  |  | 458 | 0.901 | 0.4287 | Yes |
| 61 | LSM3 |  |  | 468 | 0.892 | 0.4328 | Yes |
| 62 | ELAVL3 |  |  | 473 | 0.888 | 0.4378 | Yes |
| 63 | AKAP12 |  |  | 487 | 0.878 | 0.4410 | Yes |
| 64 | PNMA2 |  |  | 492 | 0.875 | 0.4460 | Yes |
| 65 | DACH1 |  |  | 521 | 0.851 | 0.4461 | Yes |
| 66 | FZD3 |  |  | 537 | 0.845 | 0.4487 | Yes |
| 67 | NCAN |  |  | 540 | 0.842 | 0.4538 | Yes |
| 68 | BIRC5 |  |  | 541 | 0.842 | 0.4593 | Yes |
| 69 | SBK1 |  |  | 548 | 0.833 | 0.4636 | Yes |
| 70 | CXCR4 |  |  | 550 | 0.830 | 0.4688 | Yes |
| 71 | TMTC4 |  |  | 553 | 0.828 | 0.4739 | Yes |
| 72 | KIF1A |  |  | 572 | 0.817 | 0.4757 | Yes |
| 73 | MSH6 |  |  | 593 | 0.804 | 0.4771 | Yes |
| 74 | CYFIP2 |  |  | 621 | 0.788 | 0.4769 | Yes |
| 75 | EVL |  |  | 623 | 0.787 | 0.4819 | Yes |
| 76 | PLPPR5 |  |  | 638 | 0.780 | 0.4843 | Yes |
| 77 | PBX3 |  |  | 657 | 0.771 | 0.4858 | Yes |
| 78 | EEF1A2 |  |  | 660 | 0.769 | 0.4905 | Yes |
| 79 | C3orf14 |  |  | 662 | 0.769 | 0.4953 | Yes |
| 80 | SCG2 |  |  | 682 | 0.756 | 0.4965 | Yes |
| 81 | SOX11 |  |  | 690 | 0.751 | 0.5001 | Yes |
| 82 | DPYSL3 |  |  | 691 | 0.750 | 0.5050 | Yes |
| 83 | ANP32A |  |  | 707 | 0.743 | 0.5069 | Yes |
| 84 | GRIA2 |  |  | 713 | 0.741 | 0.5108 | Yes |
| 85 | FIGNL1 |  |  | 714 | 0.741 | 0.5157 | Yes |
| 86 | ADAM22 |  |  | 716 | 0.740 | 0.5203 | Yes |
| 87 | ENO2 |  |  | 728 | 0.731 | 0.5230 | Yes |
| 88 | BMP7 |  |  | 777 | 0.711 | 0.5182 | Yes |
| 89 | MMD |  |  | 806 | 0.697 | 0.5173 | Yes |
| 90 | FHOD3 |  |  | 820 | 0.691 | 0.5193 | Yes |
| 91 | PPP1R9A |  |  | 825 | 0.688 | 0.5230 | Yes |
| 92 | C14orf132 |  |  | 834 | 0.686 | 0.5259 | Yes |
| 93 | KLHL13 |  |  | 853 | 0.677 | 0.5269 | Yes |
| 94 | TFAP2B |  |  | 884 | 0.666 | 0.5253 | Yes |
| 95 | ZNF711 |  |  | 889 | 0.663 | 0.5289 | Yes |
| 96 | CCSAP |  |  | 909 | 0.656 | 0.5295 | Yes |
| 97 | RNF165 |  |  | 927 | 0.649 | 0.5304 | Yes |
| 98 | ST3GAL6 |  |  | 941 | 0.646 | 0.5321 | Yes |
| 99 | TMEM97 |  |  | 963 | 0.636 | 0.5321 | Yes |
| 100 | RBP1 |  |  | 984 | 0.624 | 0.5323 | Yes |
| 101 | RANBP1 |  |  | 999 | 0.620 | 0.5336 | Yes |
| 102 | EIF1B |  |  | 1000 | 0.620 | 0.5377 | Yes |
| 103 | LSM4 |  |  | 1003 | 0.619 | 0.5414 | Yes |
| 104 | GABRB3 |  |  | 1044 | 0.610 | 0.5375 | No |
| 105 | ZWILCH |  |  | 1048 | 0.609 | 0.5409 | No |
| 106 | RTN1 |  |  | 1100 | 0.592 | 0.5348 | No |
| 107 | RNF157 |  |  | 1114 | 0.589 | 0.5361 | No |
| 108 | TMOD2 |  |  | 1119 | 0.588 | 0.5392 | No |
| 109 | PEG3 |  |  | 1153 | 0.578 | 0.5365 | No |
| 110 | ZNF91 |  |  | 1202 | 0.561 | 0.5308 | No |
| 111 | AP1S2 |  |  | 1205 | 0.560 | 0.5341 | No |
| 112 | PARP6 |  |  | 1235 | 0.550 | 0.5320 | No |
| 113 | GLDC |  |  | 1245 | 0.547 | 0.5338 | No |
| 114 | PRSS12 |  |  | 1250 | 0.546 | 0.5366 | No |
| 115 | CSE1L |  |  | 1259 | 0.543 | 0.5386 | No |
| 116 | CCDC167 |  |  | 1303 | 0.531 | 0.5337 | No |
| 117 | GDI1 |  |  | 1330 | 0.526 | 0.5320 | No |
| 118 | ELAVL2 |  |  | 1338 | 0.523 | 0.5341 | No |
| 119 | IRS2 |  |  | 1400 | 0.505 | 0.5254 | No |
| 120 | GGCT |  |  | 1414 | 0.501 | 0.5262 | No |
| 121 | LEPROTL1 |  |  | 1421 | 0.498 | 0.5282 | No |
| 122 | SHC3 |  |  | 1469 | 0.488 | 0.5222 | No |
| 123 | ANK2 |  |  | 1473 | 0.487 | 0.5248 | No |
| 124 | RAB6B |  |  | 1485 | 0.484 | 0.5259 | No |
| 125 | TH |  |  | 1503 | 0.479 | 0.5257 | No |
| 126 | NELFCD |  |  | 1530 | 0.474 | 0.5237 | No |
| 127 | ABCA3 |  |  | 1535 | 0.473 | 0.5260 | No |
| 128 | SLC35G2 |  |  | 1560 | 0.468 | 0.5244 | No |
| 129 | RNFT2 |  |  | 1573 | 0.464 | 0.5251 | No |
| 130 | GDAP1 |  |  | 1610 | 0.458 | 0.5210 | No |
| 131 | CLASP2 |  |  | 1635 | 0.451 | 0.5193 | No |
| 132 | SNAP25 |  |  | 1638 | 0.450 | 0.5218 | No |
| 133 | TUBB4B |  |  | 1659 | 0.444 | 0.5208 | No |
| 134 | TSPAN7 |  |  | 1669 | 0.442 | 0.5220 | No |
| 135 | GLCCI1 |  |  | 1764 | 0.425 | 0.5063 | No |
| 136 | FSD1 |  |  | 1766 | 0.425 | 0.5089 | No |
| 137 | UCP2 |  |  | 1779 | 0.422 | 0.5093 | No |
| 138 | PPP2R3C |  |  | 1800 | 0.418 | 0.5081 | No |
| 139 | NET1 |  |  | 1801 | 0.417 | 0.5109 | No |
| 140 | KIF2A |  |  | 1857 | 0.409 | 0.5028 | No |
| 141 | MANEAL |  |  | 1901 | 0.403 | 0.4970 | No |
| 142 | REEP1 |  |  | 1905 | 0.403 | 0.4991 | No |
| 143 | CEP44 |  |  | 1918 | 0.401 | 0.4993 | No |
| 144 | RALGDS |  |  | 1950 | 0.396 | 0.4959 | No |
| 145 | ACOT7 |  |  | 1967 | 0.394 | 0.4953 | No |
| 146 | NMNAT2 |  |  | 1970 | 0.393 | 0.4975 | No |
| 147 | CRMP1 |  |  | 1973 | 0.392 | 0.4997 | No |
| 148 | HNRNPA0 |  |  | 1983 | 0.391 | 0.5005 | No |
| 149 | TUBB3 |  |  | 1999 | 0.388 | 0.5001 | No |
| 150 | PPM1E |  |  | 2040 | 0.382 | 0.4947 | No |
| 151 | MSI2 |  |  | 2042 | 0.382 | 0.4970 | No |
| 152 | PHF21B |  |  | 2053 | 0.380 | 0.4976 | No |
| 153 | SEPT3 |  |  | 2062 | 0.378 | 0.4985 | No |
| 154 | TMEM108 |  |  | 2114 | 0.372 | 0.4909 | No |
| 155 | KIAA1211 |  |  | 2131 | 0.370 | 0.4902 | No |
| 156 | SATB1 |  |  | 2251 | 0.356 | 0.4692 | No |
| 157 | ATP6V1B2 |  |  | 2344 | 0.342 | 0.4535 | No |
| 158 | CAMSAP1 |  |  | 2376 | 0.338 | 0.4496 | No |
| 159 | ZNF22 |  |  | 2452 | 0.328 | 0.4371 | No |
| 160 | TMEM178B |  |  | 2521 | 0.320 | 0.4258 | No |
| 161 | FAM107B |  |  | 2546 | 0.317 | 0.4232 | No |
| 162 | NBEA |  |  | 2562 | 0.315 | 0.4223 | No |
| 163 | AUTS2 |  |  | 2613 | 0.309 | 0.4146 | No |
| 164 | RUFY3 |  |  | 2710 | 0.298 | 0.3977 | No |
| 165 | RIMBP2 |  |  | 2735 | 0.297 | 0.3950 | No |
| 166 | DUSP4 |  |  | 2799 | 0.289 | 0.3845 | No |
| 167 | RGS17 |  |  | 2930 | 0.276 | 0.3608 | No |
| 168 | LRRTM2 |  |  | 2973 | 0.272 | 0.3544 | No |
| 169 | SLC10A4 |  |  | 3015 | 0.267 | 0.3481 | No |
| 170 | AGTPBP1 |  |  | 3054 | 0.263 | 0.3424 | No |
| 171 | ADGRB3 |  |  | 3066 | 0.262 | 0.3420 | No |
| 172 | TAGLN3 |  |  | 3095 | 0.260 | 0.3382 | No |
| 173 | DNAJB1 |  |  | 3143 | 0.256 | 0.3307 | No |
| 174 | NCS1 |  |  | 3150 | 0.255 | 0.3312 | No |
| 175 | HEY1 |  |  | 3267 | -0.262 | 0.3101 | No |
| 176 | NCOA7 |  |  | 3301 | -0.266 | 0.3054 | No |
| 177 | NFIL3 |  |  | 3700 | -0.331 | 0.2296 | No |
| 178 | CYGB |  |  | 3806 | -0.350 | 0.2113 | No |
| 179 | GATA3 |  |  | 4015 | -0.392 | 0.1732 | No |
| 180 | MRPL48 |  |  | 4188 | -0.434 | 0.1423 | No |
| 181 | MAP1B |  |  | 4298 | -0.472 | 0.1240 | No |
| 182 | POPDC3 |  |  | 4522 | -0.553 | 0.0840 | No |
| 183 | CDC42EP3 |  |  | 4669 | -0.627 | 0.0595 | No |
| 184 | SEPT6 |  |  | 4793 | -0.706 | 0.0400 | No |
| 185 | C4orf48 |  |  | 4883 | -0.789 | 0.0277 | No |
| 186 | HMGA1 |  |  | 4962 | -0.878 | 0.0182 | No |
| 187 | GAL |  |  | 4967 | -0.883 | 0.0232 | No |
| 188 | NGRN |  |  | 4990 | -0.915 | 0.0249 | No |
| 189 | L1CAM |  |  | 5052 | -1.035 | 0.0197 | No |
| 190 | GLRX |  |  | 5121 | -1.212 | 0.0144 | No |
| 191 | SYT1 |  |  | 5131 | -1.248 | 0.0208 | No |
| 192 | MIAT |  |  | 5225 | -1.704 | 0.0137 | No |
Table: GSEA details [plain text format]

  

Fig 2: VANGRONINGEN\_ADRN: Random ES distribution      
 Gene set null distribution of ES for **VANGRONINGEN\_ADRN**
